# Supplementary material for: Intranasal immunization with a proteosome-adjuvanted SARS-CoV-2 spike protein-based vaccine is immunogenic and efficacious in mice and hamsters
Source: Sci Rep. 2022 Jun 13;12:9772. doi: 10.1038/s41598-022-13819-5 (PMC9191540; doi:10.1038/s41598-022-13819-5)
Supplement: Supplementary file 1 — Supplementary Figures. [file 41598_2022_13819_MOESM1_ESM.docx]

**Figure S1**: Neutralization activity induced by SmT1v3 and BDX301 vaccine formulations in mice as measured in international units. BALB/c mice (n=10/group) were immunized twice on Days 0 and 21. Vehicle control (Veh.) or the antigen (Ag) SmT1v3 (10 µg) with aluminum phosphate (Alum) (100 µg) were administered via the intramuscular (IM) route, while SmT1v3 (10 µg) with or without BDX301 (5 µg) were administered via the intranasal (IN) route. Spike protein based on the ancestral reference strain (Panel A), or Beta variant (Panel B) to VERO E6 cells. The neutralization activity is calculated as international units per millilitre (IU/mL) as related to the WHO international standard (NIBSC 20/136). For statistical analysis, values were analyzed by a one-way ANOVA with Tukey’s multiple comparisons test. ****: p<0.0001, **:p<0.01, n.s.: no significant difference.

**Figure S2:** Neutralization activity induced by SmT1v3 and BDX301 formulations in hamsters as measured in international units. Syrian Golden hamsters (n=10/group) were immunized twice on Days 0 and 21 with PBS (vehicle control, Veh.) delivered intramuscularly or BDX301 (5 µg) with or without SmT1v3 (5 µg or 15 µg) via the intranasal route. Serum collected on Day 35 was analyzed for its ability to block binding of Spike protein based on the ancestral reference strain (Panel A), Beta variant (Panel B) or Delta variant (Panel C) to VERO E6 cells. The neutralization activity is calculated as international units per millilitre (IU/mL) as related to the WHO international standard (NIBSC 20/136). For statistical analysis, values were analyzed by a one-way ANOVA with Tukey’s multiple comparisons test. **: p<0.01, *:p<0.05.
